# BOLD fMRI of C-Fiber Mediated Nociceptive Processing in Mouse Brain in Response to Thermal Stimulation of the Forepaws

**DOI:** 10.1371/journal.pone.0126513

**Published:** 2015-05-07

**Authors:** Simone C. Bosshard, Florian Stuker, Constantin von Deuster, Aileen Schroeter, Markus Rudin

**Affiliations:** 1 Institute for Biomedical Engineering, University and ETH Zurich, Zurich, Switzerland; 2 Center for Neurosciences, University and ETH Zürich, Zurich, Switzerland; 3 Institute of Pharmacology and Toxicology, University of Zurich, Zurich, Switzerland; Toronto University, CANADA

## Abstract

Functional magnetic resonance imaging (fMRI) in rodents enables non-invasive studies of brain function in response to peripheral input or at rest. In this study we describe a thermal stimulation paradigm using infrared laser diodes to apply noxious heat to the forepaw of mice in order to study nociceptive processing. Stimulation at 45 and 46°C led to robust BOLD signal changes in various brain structures including the somatosensory cortices and the thalamus. The BOLD signal amplitude scaled with the temperature applied but not with the area irradiated by the laser beam. To demonstrate the specificity of the paradigm for assessing nociceptive signaling we administered the quaternary lidocaine derivative QX-314 to the forepaws, which due to its positive charge cannot readily cross biological membranes. However, upon activation of TRPV1 channels following the administration of capsaicin the BOLD signal was largely abolished, indicative of a selective block of the C-fiber nociceptors due to QX-314 having entered the cells via the now open TRPV1 channels. This demonstrates that the cerebral BOLD response to thermal noxious paw stimulation is specifically mediated by C-fibers.

## Introduction

Functional magnetic resonance imaging (fMRI) in animals enables non-invasive studies of brain function, e.g. involving the sensory system. Electrical stimulation is a widely used stimulation paradigm for such studies [1–5] and has recently been applied to analyze sensory and nociceptive processing in mice [6,7]. However, electrical stimulation is not physiological and faces some drawbacks: 1) needle electrodes are inserted subcutaneously into the skin, inducing a stimulus even in the absence of an electric current. 2) The stimulus strength is determined by the local current density, which depends on the (relative) placement of the electrodes. 3) The electrical current may activate the neurons directly instead of only inducing a peripheral stimulus, which then would lead to the activation of neurons. In view of these potential drawbacks, at the aim of this study was to establish a more physiological stimulation paradigm with better controllable parameters using an infrared laser to induce noxious stimulation by local heating of the forepaws. Thermal stimulation in mouse fMRI should provide a powerful tool to investigate various pathologies and mechanisms of pain disorders, taking advantage of the many available transgenic mouse models. It has the additional benefit of being a translational method, as laser heat stimulation has also been used in clinical fMRI studies [8]. This protocol led to reproducible changes in the blood oxygen level dependent (BOLD) signal intensity in the various brain areas including those associated with pain processing, with signal amplitudes increasing as a function of skin temperature.

Pain perception caused by heat is mediated by receptors of the transient receptor potential (TRP) family, the most important being the vanilloid receptor TRPV1, which is activated by temperatures of 42°C and above [9] and located on C-fiber afferents [10]. Other members of the TRP family respond to similar (TRPV3, activation threshold: 33–39°C) or higher (TRPV2, activation: 52°C) temperatures [11,12]. TRPV2 channels were reported to activate thinly myelinated Aδ-fibers [13,14], whereas TRPV3 channels are only located in keratinocytes in mice [12], but not in dorsal root ganglia (DRGs).

To demonstrate the specificity of the paradigm for assessing nociceptive signaling we applied the quaternary lidocaine derivative QX-314 (lidocaine N-ethyl chloride) to the forepaws prior to thermal stimulation. QX-314 carries a positive charge and is therefore not able to cross cell membranes. The compound has no effect on sodium channels when applied extracellularly, but was found to block sodium channels when administered intracellularly, leading to local anesthesia [15–18]. Binshtok and colleagues established a method to selectively block nociceptive signaling by activating TRPV1 channels [19]. Also known as capsaicin receptor, TRPV1 is located on C-fiber nociceptors and activated by a wide range of physical and chemical stimuli, among them capsaicin [9]. Capsaicin activates and opens the TRPV1 channel, allowing the QX-314 molecule to enter the cell and block the sodium channels by binding to a specific site located at its intracellular domain, inhibiting the propagation of action potentials [19]. In this fMRI study we showed that pretreating mice with QX-314 in combination with capsaicin led to abolishment of the BOLD fMRI signal elicited by thermal stimulation, while administration of either compound alone did not affect the signal amplitude.

## Methods

### Animal Preparation

All experiments were performed in accordance with the Swiss law of animal protection. The protocol was approved by the veterinary office of Zurich, Switzerland (Permit number ZH 173–2008, ZH 187–2011).

20 female C57Bl/6 mice weighing 22 ± 3 g were anesthetized with isoflurane (induction 2–3%, maintenance 1.2% in a 70% air—30% oxygen mixture; Abbott, Cham, Switzerland), endotracheally intubated and mechanically ventilated (90 breaths/minute, respiration cycle: 25% inhalation, 75% exhalation; MRI-1, CWE, Ardmore, PA, USA) throughout the entire experiment. This ensured maintenance of stable physiology reflected by measurements of heart rate and blood oxygenation, which was monitored using a MR-compatible infrared sensor (MouseOx Pulse Oximeter, Starr Life Sciences, Oakmont, PA, USA) in n = 20 animals, which were complemented by assessment of blood pCO_2_ levels as controlled by a transcutaneous electrode (TCM4, Radiometer, Copenhagen, Denmark), which was placed on the shaved upper hind limb of the mouse to measure blood gas levels (pCO_2_) in n = 16 of these 20 animals. A rectal temperature probe (MLT415, AD Instruments, Spechbach, Germany) was inserted to control and keep the body temperature at 36.5 ± 0.5°C, which was maintained using a warm-water circuit integrated into the animal support (Bruker BioSpin AG, Fällanden, Switzerland). Animals were paralyzed by intravenous (i.v.) administration of a neuromuscular blocking agent (Pancuronium bromide, 1.0–1.5 mg/kg; Sigma-Aldrich, Steinheim, Germany), which avoided interference by spontaneous breathing and prevented movement artifacts during the fMRI experiments despite the low isoflurane levels. Non-invasive monitoring of the mice showed stable physiology throughout the experiments. Body temperature was kept stable at 36.5 ± 0.5°C throughout the experiment. Heart rate was stable around 500 beats per minute in all animals monitored and did not change during stimulation (n = 10, n_exp_ = 19). Arterial oxygen saturation was > 97% and pCO2 levels were in the range 35–40 mmHg indicating a well-adjusted ventilation of the animals [20]. After completion of the fMRI experiments, the animals recovered fast and were able to be used for further experiments, after a resting period of at least 2 weeks.

### Experimental Groups

For the implementation and optimization of the thermal stimulation paradigm three conditions were evaluated: *Group 1* Stimulation at 45°C using a 2 mm diameter laser spot (n_animals_ = 10, n_scans_ = 19). *Group 2* Stimulation at 46°C using a 2 mm diameter laser spot (n_animals_ = 6, n_scans_ = 11). *Group 3* Stimulation at 46°C using a laser spot of 1 mm in diameter (n_animals_ = 6, n_scans_ = 12). Higher temperatures were not used in order to avoid skin burns.

Pharmacological modulation of the thermal stimulation was used to evaluate the specificity of the protocol. For optimal sensitivity, parameters prompting a robust BOLD signal were used (corresponding to 45°C using a 2 mm diameter laser spot). Again three groups of animals were studied: *Group 4* Animals receiving an injection of 10 μl of a solution containing 67 mM QX-314 and 1.6 mM capsaicin locally into the left and right forepaw 80 and 100 min before thermal stimulation, respectively (n_animals_ = 7, n_scans_ = 14). The mixture was prepared from stock solutions of 5 μl QX-314 (134 mM, lidocaine N-ethyl chloride, Sigma-Aldrich, Steinheim, Germany) dissolved in 0.9% NaCl and 5 μl capsaicin (3.3 mM, Sigma-Aldrich, Steinheim, Germany) dissolved in ethanol and diluted with 0.9% NaCl.

Two control groups were used: *Group 5* Animals receiving 10 μl of 67 mM QX-314 in 0.9% NaCl into the left and right forepaw 80 and 100 minutes prior to thermal stimulation, respectively (n_animals_ = 3, n_scans_ = 6), and *Group 6* mice receiving10 μl solution containing 1.6 mM capsaicin dissolved in ethanol and 0.9% NaCl into the left and right forepaw 80 and 100 minutes before stimulation (n_animals_ = 3, n_scans_ = 6).

To minimize the number of animals being used in this study, we reused all animals from groups 1, 2 and 3 for further experiments. After a recovery period lasting at least two weeks, they were used for a second experiment in either group 3, 4, 5 or 6.

### Thermal Stimulation

**Fig 1:**
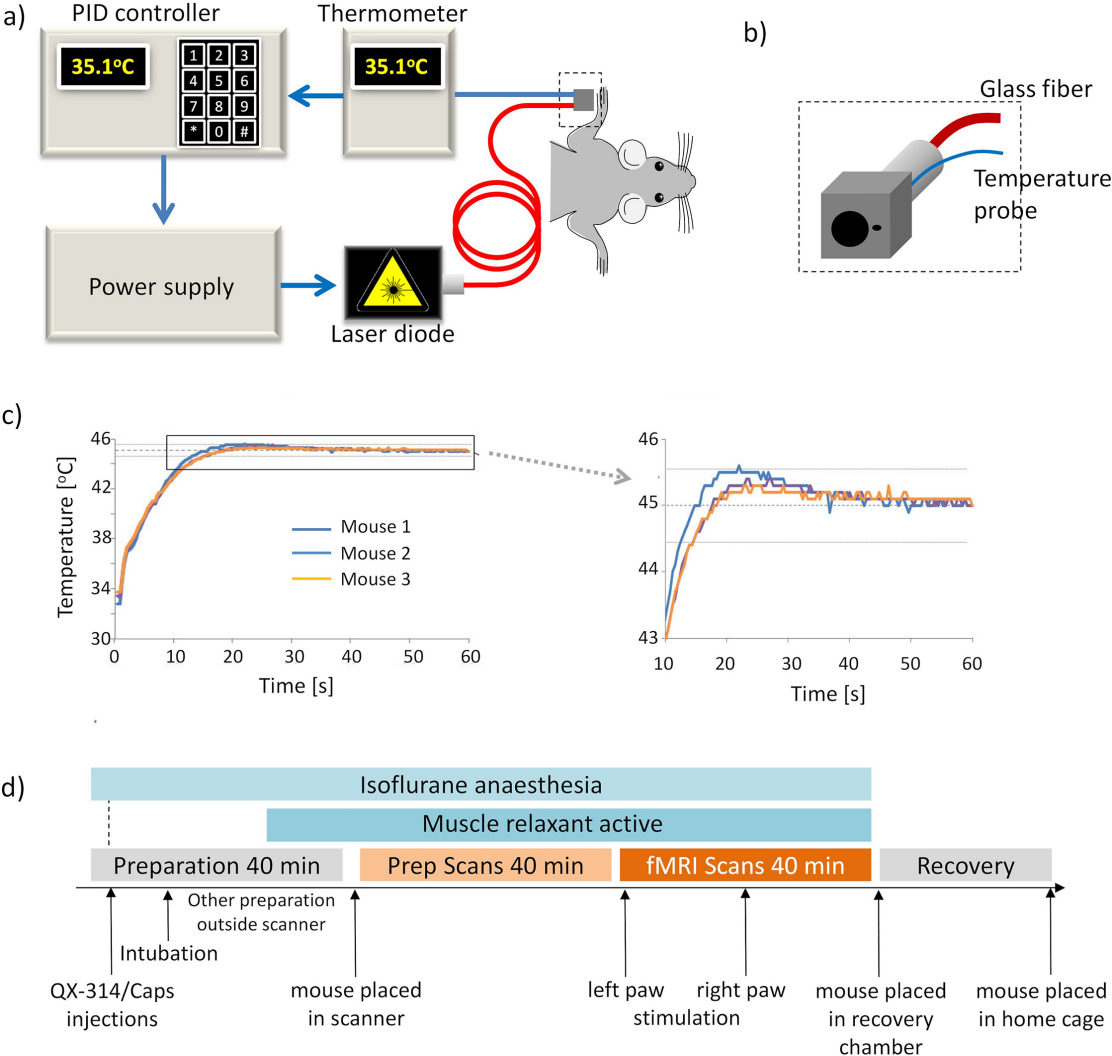
Thermal stimulation setup. **(a)** Scheme of setup of the laser stimulation with feedback loop for temperature control. **(b)** Close-up view of the cube for paw fixation made from black Perspex. Two hole diameters were used, enabling irradiation at spot sizes of 1 and 2mm, respectively. The thermocouple used for temperature monitoring was positioned immediately adjacent to the irradiated area of the paw. **(c)** Temperature profiles during stimulation, recorded at the mouse forepaw for three different animals. Target temperature of 45 ± 0.5°C was reached for at least 30 s. d) Experimental protocol for fMRI studies.

Thermal stimulation was performed using a custom built stimulation device consisting of two 8 Watt infrared laser diodes operating at 975 nm (BMU8_975_01_R, Oclaro, San Jose, CA, USA), which were connected to 5 m glass fibers (Thorlabs Inc., München, Germany) and guided into the Faraday cage of the scanner through cylindrical radiofrequency stacks. The power output of the laser was regulated by a custom built power supply (Meerstetter Engineering GmbH, Rubigen, Switzerland). Two types of cubes made from black Perspex with a small hole drilled in the center (1 or 2 mm) were mounted on the SMA connector at the end of the glass fibers, allowing choosing between two different diameters of the laser spot (1 or 2 mm) (Fig 1B). In addition, a temperature probe was placed next to the hole to record the temperature of the paw (Fig 1B). The temperature probe was connected to a thermoelement (P600, Dostman Electronic, Wertheim-Reicholzheim, Germany). A homebuilt proportional-integral-derivative (PID) controller was used as a feedback control, regulating the laser power supply in order to maintain the temperature measured at the paw at the set target temperature. On/off cycles as defined by the stimulation paradigm were controlled from a physiological monitoring device (Powerlab, ADInstruments, Spechbach, Germany), sending a trigger pulse to the PID controller (Fig 1A).

The stimulation paradigm consisted of a block design starting with a resting period of 120 s (off, baseline) followed by 60 s of stimulation (on). This series was repeated four times and fMRI data acquisition was continued for another seven minutes after the last stimulation block. A stimulation experiment was considered successful if the target temperature at the paw was maintained for at least 30 s with an accuracy of ± 0.5°C. Stimulation started with the left paw in all animals. Following a resting period of 8 minutes, the right paw was stimulated.

The feedback controlled temperature regulation of the laser worked reliably for both spot diameters. On average, the target temperature was reached after 20–25 s and maintained for the remainder of the stimulation period with a maximal variation of ± 0.5°C (Fig 1C). Only 2 out of 64 scans had to be discarded because the target temperature could not be reached within 30 s.

### MRI Equipment and Sequences

All experiments were carried out on a Bruker BioSpec 94/30 small animal MR system (Bruker BioSpin MRI, Ettlingen, Germany) operating at 400 MHz (9.4 Tesla). For signal transmission and reception a commercially available cryogenic quadrature RF surface probe operating at 30 K was used (Bruker BioSpin AG, Fällanden, Switzerland) [21]. The ceramic outer surface of the coil touching the mouse head was kept at 30°C using a temperature-controlled heating device.

Anatomical reference images in coronal and sagittal directions (slice orientations are given using the nomenclature of the mouse brain atlas [22]) were acquired using a spin-echo (Turbo-RARE) sequence (for detailed parameters see [6]). Subsequently, the slices for the fMRI experiment were planned on the anatomical reference images and BOLD fMRI data were acquired using a gradient-echo echo planar imaging (GE-EPI) sequence with the following parameters: Five coronal slices covering a range of 2 to 5 mm anterior to the interaural line were recorded with a field-of-view FOV = 23.7 x 12.0 mm^2^, matrix dimension MD = 90 x 60 (acquisition) and 128 x 64 (reconstruction), yielding an in-plane resolution of 200 x 200 μm^2^, slice thickness STH = 0.5 mm, interslice distance ISD = 0.7 mm (0.2 mm gap between slices). For the optimization experiments (1) and (2), values for repetition time TR = 2500 ms, echo time TE = 8.5 ms, number of averages NA = 3, and number of repetitions NR = 152 were used, resulting in an image acquisition time of 7.5 seconds. For all remaining experiments, the parameters were as follows: TR = 1000 ms, TE = 8.5 ms, NA = 1 and NR = 1140, resulting in a temporal resolution of 1 second. All fMRI acquisitions lasted 19 min. The slices were placed based on anatomical landmarks, which allowed reproducible positioning and images in all animals.

### Data Analysis and Statistics

Data analysis was performed using the Biomap software program (M. Rausch, Novartis Institute for Biomedical Research, Switzerland). No preprocessing or normalization of the image data were carried out. Statistical t-maps were calculated using the general linear model (GLM) tool, which assesses correlations on a voxel-by-voxel basis between the fMRI signal train and the stimulation paradigm. Activation was detected using a statistical threshold of p = 0.0001 for all experiments and a minimal cluster size of 15 voxels. The respective regions-of-interest (ROIs) derived from the GLM analyses were used to extract the BOLD signal changes as a function of time. In cases for which the correlation analysis revealed no activated voxels at the expected locations, ROIs were transferred from the mouse brain atlas [22]. Data analysis was performed for the key regions of nociceptive processing: the primary and secondary somatosensory cortices (S1, S2), and the thalamus. In addition to these regions, a control region not involved in nociceptive processing was evaluated.

GLM-derived activation patterns (EPI images covering the S1 area (IAL +3.7 mm)) were used for group analysis [6]. The EPI images were normalized to the coordinate system of the mouse brain atlas [22]. The fMRI coordinates were defined as followed: the origin of the right-hand coordinate system was chosen at the ventral end of the brain midline through the coronal sections. The second reference point was the dorsal end of the same midline, while the third point was placed on the edge of the right hemisphere at its widest point. The coordinate axes were defined along the midline (y-axis) and perpendicular to it (x-axis). The axes were then scaled to fit the dimensions of the mouse brain atlas, using an IDL-based software developed in-house [23].

Comparative statistics was performed taking the maximal BOLD value of the first stimulation period of each animal (Origin 7.5, OriginLab Corp., Northampton, MA, USA). Values were not normally distributed and therefore tested at the α = 0.05 level using the non-parametric Kruskal-Wallis test followed by the *post hoc* Bonferroni test (comparison between different groups). All values are presented as mean ± SEM.

The decay rate of the BOLD signal, obtained by single exponential fitting of the first 40 s following the end of the first stimulation period, was correlated with the amount of heat to be dissipated. The heat deposited in tissue that had to be dissipated, was estimated according to ∆Q = c_p_ · ∆T · ρ · (π · r^2^) · d with c_p_ = heat capacity, ∆T = temperature difference of T_mean_-T_thresh_ and r = spot radius. T_thresh_ is the temperature required to elicit a pain response (42°C). The heat capacity of tissue was assumed c_p_ = 4 [J·K^-1^·g^-1^], the density ρ = 1 g/cm^3^ and the thickness of the affected tissue d = 0.5 mm. As these parameters appear as linear factors in the heat equation, any errors in estimation will not affect the accuracy of the fit, just the scaling of the abscissa.

## Results

### BOLD Signal Changes Correlate with the Thermal Stimulation Paradigm

**Fig 2:**
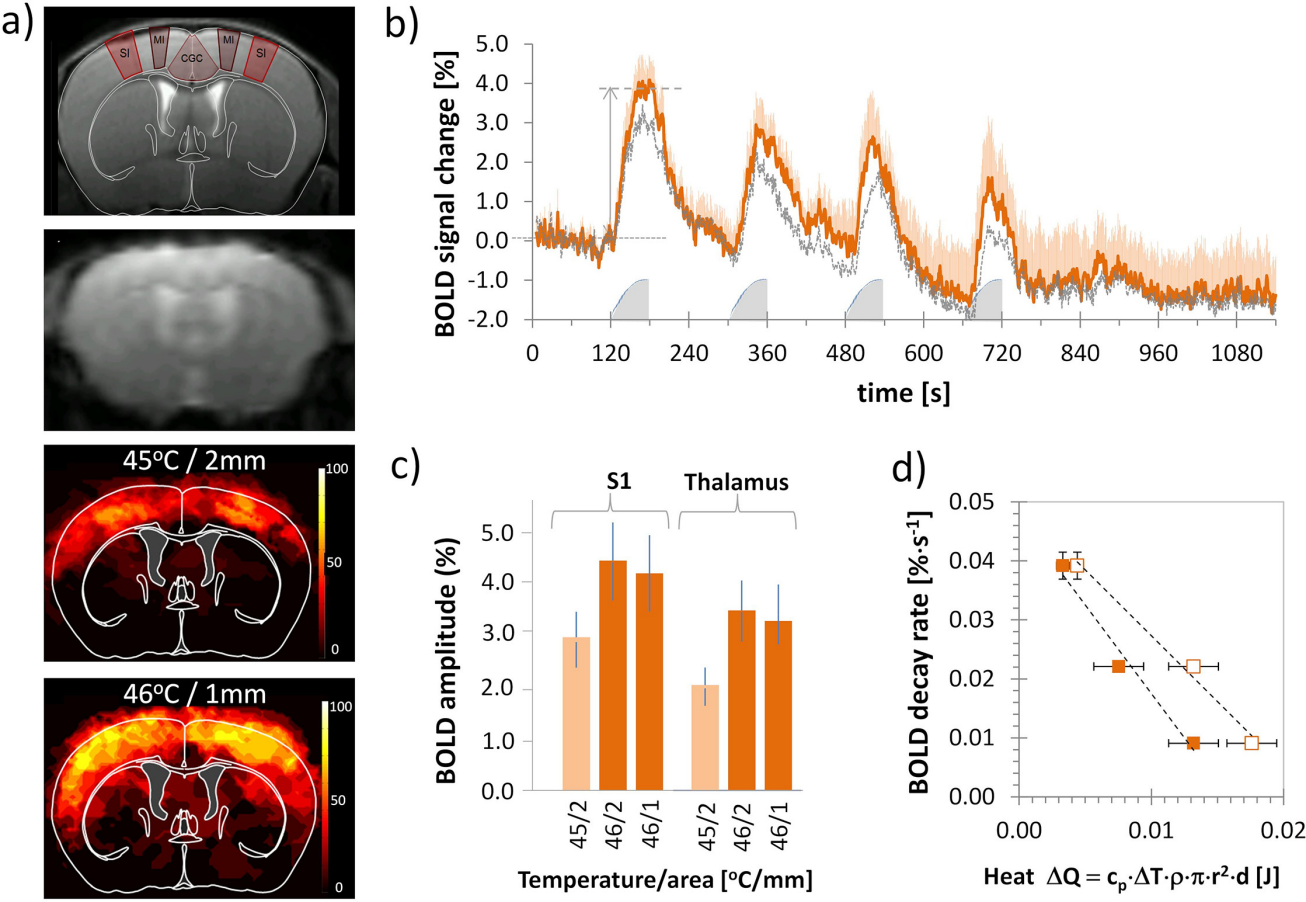
BOLD signal changes induced by thermal stimulation. **(a)** Anatomical MR image (top panel) with overlay of respective section from mouse brain atlas (IAL +3.7 mm). Regions relevant for pain processing are indicated (SI: primary somatosensory cortex, forepaw region; MI: primary motor cortex; CGC: cingulated cortex) and representative EPI image (second panel). Combined group activation maps after left and right thermal forepaw stimulation of 45 (n_scans_ = 19, third panel) and 46°C (n_scans_ = 12, bottom panel) show activated regions derived from GLM analysis (p = 0.0001, cluster size 15 voxels) for all animals overlaid on the mouse brain atlas. The scale bar indicates the percentage of animals showing significant BOLD activation at the given threshold. **(b)** Mean temporal BOLD profile of the somatosensory cortex (S1; red with error bars) and thalamus (dashed gray; without error bars) contralateral to the stimulated paw (n_scans_ = 12, orange). Stimulation parameters: 46°C, 1 mm. Grey shaded blocks indicate stimulation periods. Arrow indicates amplitude measure for quantitative analysis (for somatosensory cortex S1). **(c)** Maximum BOLD signal amplitude of first stimulation period for S1 and thalamus for T = 45°C/2 mm, T = 46°C/2 mm, and T = 46°C/1 mm. **(d)** Decay rate of BOLD signal as a function of heat dissipated. There is a linear correlation between the decay rate and the amount of ‘noxious`heat (T_thresh_ = 42°C, R^2^ = 0.988, open symbols) and (T_thresh_ = 43°C, R^2^ = 0.974, filled symbols) deposited in the tissue. All values are given as mean ± SEM.

Thermal stimulation of the forepaws led to consistent BOLD responses in various brain regions including the S1 and S2 somatosensory cortices (Fig 2A) and the thalamus. The signal changes correlated well with the stimulation pattern and intensity (Fig 2A and 2B). The image and signal quality was high even when increasing the temporal resolution to 1 second.

The maximal BOLD signal change at 45°C was 2.8 ± 0.5% in the S1 area contralateral to the stimulated paw and 1.8 ± 0.4% in the thalamus. At 46°C, the maximal BOLD signal changes in the contralateral S1 area were 4.4 ± 0.9% and 4.1 ± 0.6% for the laser spots of 2 mm and 1 mm in diameter, respectively. The corresponding maximal BOLD signal changes in the thalamus were 3.3 ± 1.0% (2 mm diameter) and 3.1 ± 0.6% (1 mm diameter) (Fig 2C). The BOLD amplitude was influenced by the stimulation temperature, but not by the diameter of the laser spot (Fig 2C). On the other hand it was found that the decay rate of the BOLD signal following the stimulation interval increased with increasing spot diameter. An excellent correlation (R^2^ = 0.9876) was observed between the rate of post-stimulus BOLD signal decay and the amount of noxious heat (assuming threshold temperatures of T_thresh_ = 42°C or 43°C, respectively) deposited in the tissue (Fig 2D).

### Nociceptive Block Induced by QX-314 and Capsaicin

**Fig 3:**
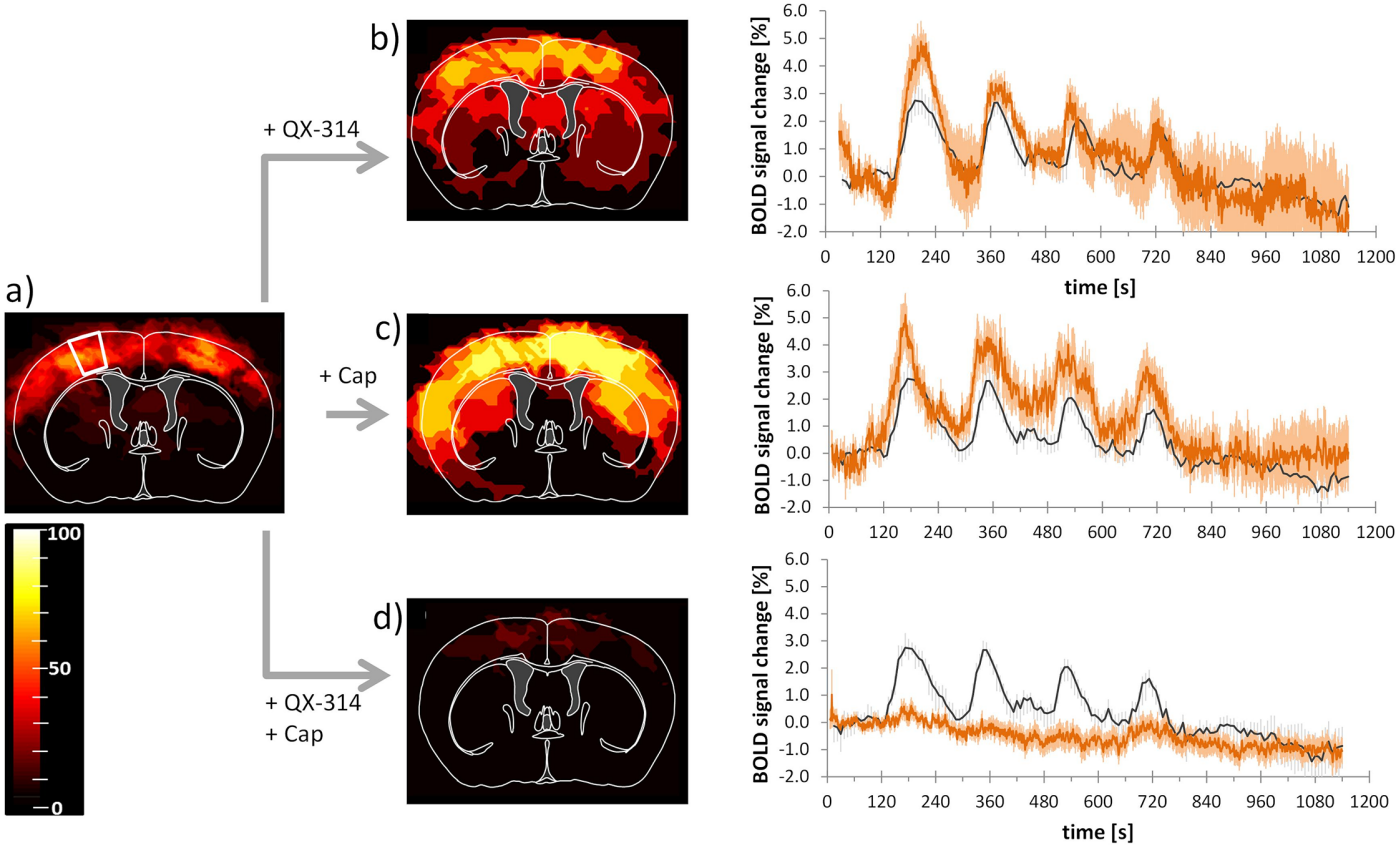
Pretreatment with capsaicin and QX-314 abolishes BOLD response. Activation maps and BOLD signal profiles after left and right thermal forepaw stimulation at 45°C. **(a)** Control condition, thermal stimulation of naïve animals. The white outline indicates the area used for extracting BOLD signal profiles. **(b)** After pretreatment with QX-314 (n_scans_ = 6); **(c)** after pretreatment with capsaicin (Cap, n_scans_ = 6,); and **(d)** after pretreatment with QX-314 and capsaicin (n_scans_ = 14). Images show activated regions derived from GLM analysis (p = 0.0001, cluster size 15 voxels) for all animals overlaid on the mouse brain atlas. The scale bar indicates the percentage of animals showing significant BOLD activation at the given threshold. Profiles show BOLD response for individual treatments (red). For reference, the profiles of control (naïve) animals are indicated in dark grey. **(b-d)**. All values are given as mean ± SEM.

The group analysis of all animals reflects the BOLD signal changes after thermal stimulation and treatment with QX-314 and/or capsaicin. The activity maps show the main activation appearing in the S1 area after stimulation of both paws at 45°C (Fig 3). Pretreatment with QX-314 combined with capsaicin only led to cortical activation in two of 14 scans (Fig 3D). Pretreatment of either compound alone did not diminish the activation, but rather increased the activated areas in the brain, though the effects were not significant.

**Fig 4:**
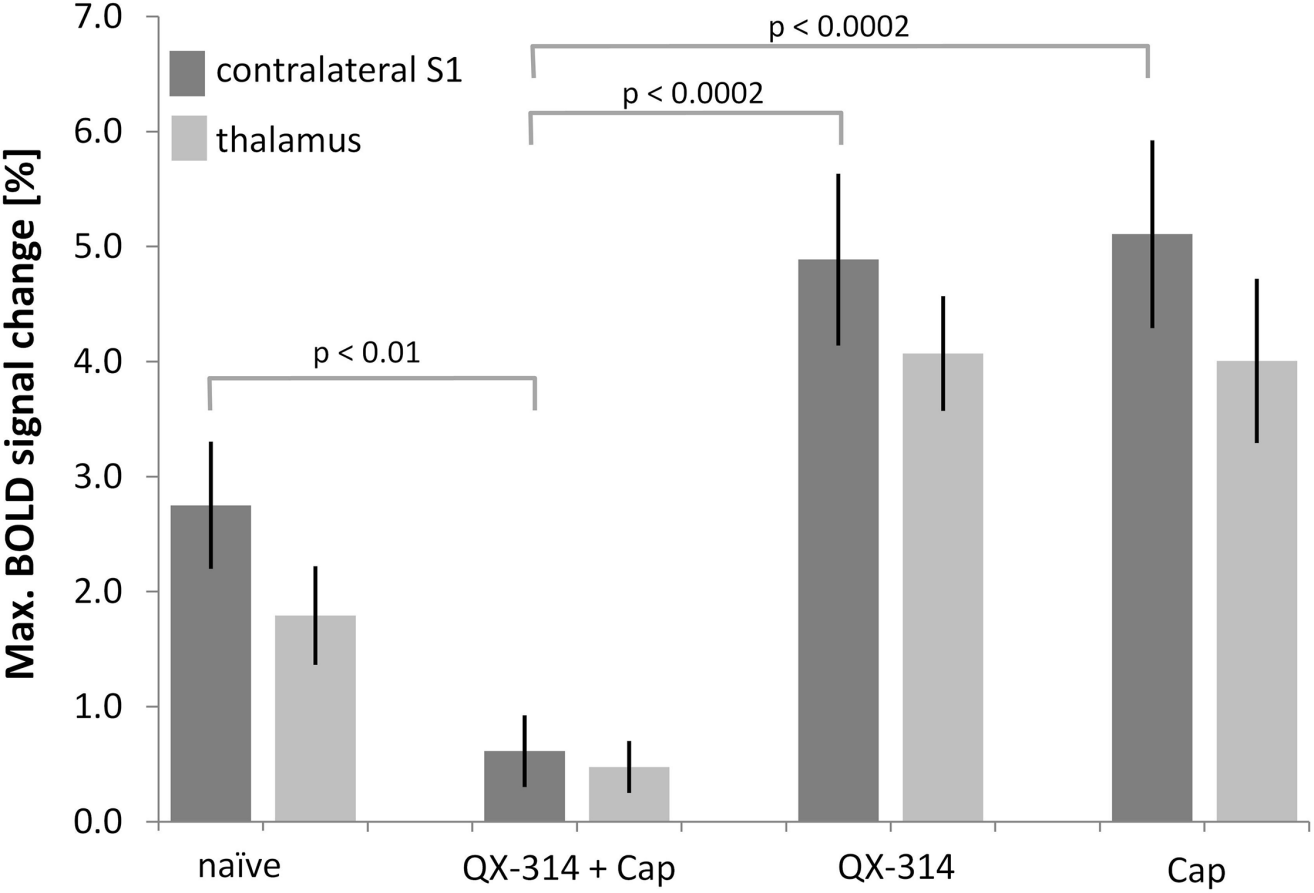
Pretreatment with capsaicin and QX-314 abolishes BOLD response. **(a)** Maximum BOLD signal changes of the S1 contralateral to the stimulated paw and the thalamus. Pretreatment with QX-314 and capsaicin (Cap) led to an abolishment of the BOLD signal, while treatment with either substance alone did not decrease the BOLD signal change. All values are given as mean ± SEM. For clarity, only p-values for the S1 region are displayed. All p-values are given in the text.

Combined application of the lidocaine derivative QX-314 and capsaicin led to a decreased BOLD activation detected in the brain (S1: 0.6 ± 0.3%, p = 0.01; thalamus: 0.5 ± 0.2%, p = 0.08; Figs 3D, 4) indicative of a specific inhibition of neuronal signal transmission via C-fibers. This inhibitory effect was not observed in the control experiments with either compound applied separately. Administration of QX-314 alone led to a maximal BOLD signal change of 4.9 ± 0.7% in the S1 (n_scans_ = 6, Figs 3B, 4), which was not significantly different from the untreated animals (p = 0.15), but significantly different from the combination treatment capsaicin plus QX-314 (p = 0.0002, n_scans_ = 14, Figs 3D, 4). The maximum BOLD intensity of the thalamus after treatment with QX-314 alone (4.0 ± 0.5%) was significantly different compared with untreated mice (p = 0.02) and different from values obtained with the combination treatment (p = 0.0001) (Fig 4). Application of capsaicin alone led to maximal BOLD signal changes of 5.1 ± 0.8% and 4.0 ± 0.7% in the S1 and thalamus respectively, which were both significantly larger than the corresponding values measured after combined treatment (p = 0.0002 and p = 0.0009, respectively, n_scans_ = 6, Figs 3C, 4). However, compared with the untreated animals, only the BOLD response in the thalamus showed a significant increase (p = 0.02), while the response in the S1 area was not different (p = 0.08; Fig 3A).

## Discussion

fMRI has become an important and widely used tool in animal research, as it allows non-invasive monitoring of brain functions in large brain volumes. Multiple studies in humans and in rodents have shown a good correspondence of the noxious-evoked activation patterns detected by fMRI and the network of the pain matrix [6,7,24–28]. It is apparent that activated regions comprised the somatosensory S1 and S2, motor M1, the cingulate cortex, as well as thalamic nuclei. However, one has to keep in mind that due to the hemodynamic nature of the signal, the activated areas may not be tightly confined to the functional brain structures but include areas covered by draining vessels, which renders a strict allocation difficult. Furthermore, we observed a strict bilaterality of the fMRI response to unilateral sensory input with contra- and ipsilateral somatosensory cortices showing essentially identical BOLD signal changes in line with earlier reports [6,7]. A recent study comparing four different anesthesia paradigms has shown that sensory input, such as electrical stimulation of the hind paw, prompts a systemic hemodynamic response in anesthetized mice that may overrule the cerebral autoregulation and induce widespread bilateral activation patterns that comprise the elements of the pain matrix [29]. While this inevitably will limit the spatial specificity of the cerebral fMRI response, it will nevertheless allow assessing the changes in the BOLD signal upon modulation of the peripheral input.

A crucial point that needs to be considered, also in view of translational studies, is the use of anesthetics in animal fMRI. In contrast to human fMRI studies, where the subjects typically are conscious, most animal studies are performed in anesthetized animals in order to reduce motion artifacts and stress. All commonly used anesthetics in animal studies have one or more effects on neuronal metabolism, CBF, neurovascular coupling or sensory perception [30–33]. Artificial ventilation and paralyzing the animal with a neuromuscular blocking agent allowed maintaining stable physiological conditions and prevented motion artefacts when working at relatively low concentrations of isoflurane. At these levels (1.2%) we assume minimal antinociceptive efficacy as well as preserved neurovascular coupling [34,35]. The robust and sensitive BOLD signal changes observed confirm the suitability of the applied anesthesia protocol.

In imaging in general and in fMRI in particular, there is a trade-off between sensitivity (i.e. signal-to-noise ratio, SNR), temporal resolution and spatial resolution. In this study we have optimized a protocol [6] to improve temporal resolution from 7.5 s to 1 s, while maintaining spatial resolution and only minimally compromising SNR in order to be sensitive enough to detect signal changes of only a few percent. Increasing temporal resolution to 1 s is essential for better characterizing the hemodynamic response elicited by the stimulus and for enhancing the correlation with the stimulus.

In contrast to electrical stimulation, thermal stimulation constitutes a physiological stimulus, which directly activates the nociceptive system. Temperature exceeding a threshold value is a noxious stimulus, which can be well controlled by adjusting parameters such as the target temperature, spot diameter and stimulation duration. By choosing target temperatures of 45 or 46°C, we predominantly induce TRPV1-mediated activation of the neural system. TRPV1 receptors are activated at temperatures of 42°C and above, and the signal is transmitted via the unmyelinated C-fiber afferents [10]. Activation of the Aδ-afferents would occur by activation of the TRPV2 receptors, which have an activation threshold of 52°C [11], a temperature not considered in our stimulation paradigm. Temperatures should be kept below 50°C to avoid any skin damage on the paw. In view of our relatively long stimulation period, skin damage might occur already at lower temperatures. Therefore all experiments were carried out at a temperature of 45°C, a temperature leading to a robust BOLD signal change of 2.8 ± 0.5% in the S1 area, which was sufficient for studying the pharmacological modulation of the response and considered not to be harmful. None of the animals displayed signs of paw injury; hence the mice could be used for more than one experiment, an important prerequisite for longitudinal studies. The maximal BOLD signal changes were not comparable to two other mouse fMRI studies, which measured brain activation upon thermal stimulation of the hindpaw, using Peltier heating devices [36,37]. The reported BOLD signal changes of 0% at 45°C and 0.7% at 60°C [36] were of much smaller amplitude than the ones observed in our study. The difference might be due to the shorter stimulation period; however, the maximum BOLD signal change observed in this study appeared shortly after reaching temperatures above 43°C. Another reason could lie in the difference of the stimulation devices, as the contact heat delivers the heat more widespread, while the laser beam was focused on one small spot of the paw, which could lead to a difference in pain perception.

The temporal profile of the BOLD signal changes correlated well with the stimulation periods. The rise of the BOLD signal was slightly delayed with regard to stimulus onset, which was due to the nature of the stimulus. The baseline temperature of the paw was typically around 31°C and on average it took 20 seconds to reach the target temperature of 45 or 46°C at the paw. The time point at which heat starts to become noxious at 42–43°C was therefore only reached approximately 10 seconds after stimulus onset (Fig 1).

The BOLD signal amplitude was influenced only by the target temperature, but not by the spot diameter. However, the signal shape appeared to be influenced by the spot diameter used. For the experiments performed with the 2 mm spot the signal was found to decay at a slower rate and did not return to baseline levels within the 2 min resting period resulting in an underlying slow signal component as described earlier [6]. The temporal BOLD profile of the experiment carried out using the 1 mm spot shows faster signal decay in the initial phase after the stimulation, and the signal returns to baseline levels within 2 min. A good correlation was found between the initial decay rate of the BOLD signal and the amount of noxious heat deposited in the tissue. In general, profiles showed a biphasic decay pattern with a fast decrease of the BOLD amplitude during the first 30 s after stimulation, followed by a slower decay or stable signal, which might be due to hemodynamic effects [6]. Another characteristic feature of the BOLD signal is the decrease in signal amplitude despite ongoing stimulation. This may be due to adaptation as has been shown for dorsal root ganglia cells, which show a slow decrease in activity over 2–3 s to continuous stimulation [13]. Alternatively, the effect may be attributed to a decaying vasodilatory signal, which is subject to feedback regulation by CBF in response to a prolonged neuronal stimulus [38,39]and which has also been observed in experiments using electrical stimulation paradigms [6].

To verify the specificity of the BOLD signal readout for C-fiber mediated nociceptive processing, we pharmacologically modulated the nociceptive signal transmission. Binshtok et al. [19] demonstrated in a recent study that the cationic lidocaine analogue QX-314 was able to enter neurons through opened TRPV1 channels. Inside the cells, QX-314 blocks the sodium channels, thereby preventing the propagation of action potentials and inducing local anesthesia. Opening of the TRPV1 channels can be achieved by administration of capsaicin, a highly potent agonist. Since TRPV1 channels are only found on C-fiber afferents in the peripheral nervous system [10], the entry of QX-314 will cause a C-fiber specific nociceptive block. In contrast, uncharged local anesthetics such as lidocaine can penetrate all fiber types and upon binding to sodium channels will lead to a transient sensory and motor inhibition [40,41]. Since thermal stimulation predominantly activates C-fibers, we expected a significant decrease of the BOLD signal after application of QX-314 in combination with capsaicin. Indeed, the BOLD signal was almost totally abolished supporting the hypothesis that it reflects nociceptive processing in mice.

The anesthetic properties of QX-314 are somewhat controversial. While several studies reported the compound not being able of passing the cell membrane and thus of no anesthetic efficacy [15,16,19,42]other studies report an anesthetic effect of QX-314 comparable to that of lidocaine, but with a slower onset[43,44]. They attribute the possible cell entry of the compound to the tonic activity of the TRPV1 channels [45], since application of the TRPV1 antagonist capsazepine prevents sensory blockade induced by QX-314 [43]. Our data demonstrating inhibition of nociceptive transmission by QX-314 only in the presence of capsaicin, but not when administered a single compound, are rather in line with the hypothesis of the compound not penetrating neurons or entering them very slowly.

The application of either substance separately did not lead to a decrease of the BOLD signal. In contrast, there was a trend towards an increase of the maximal signal amplitude. After capsaicin application this may be due to a sensitization effect since both capsaicin and noxious heat act on the same receptors. It has been shown that direct activation of the TRPV1 receptor may sensitize it to other stimuli [46]. The same may apply for the positively charged molecule QX-314, as a study by Ahern et al. has shown that cations directly gate and sensitize TRPV1 channels [47]. A transient reduction of thermal response latency in rats after injection of either 67 mM QX-314 or capsaicin (1.6 mM) has also been reported [19].

In conclusion, in this study we describe the use of BOLD fMRI in mice to characterize nociceptive processing elicited by thermal stimulation of the forepaws, which was shown to be a robust and physiological stimulation paradigm. Reproducible BOLD signals were observed in brain areas attributed to nociceptive processing (S1 and S2, thalamus). The abolishment of these signals after inhibition of nociceptive signaling demonstrates the specificity of the stimulation protocol and validates the BOLD readout as a response to noxious thermal stimulation. The method is non-invasive and therefore provides a tool for longitudinal studies of nociceptive processing in normal and genetically engineered mice e.g. to investigate mechanism involved in hyperalgesia.
